# Editorial Department

**Published:** 1861-09

**Authors:** 


[For Buffalo Medical and Surgical Journal ]

MR. EDITOR:

We wish to correct through your columns a mis-statement of Dr. Bly's, in his Circular, which appeared in your Journal, that one Henry Lipps has had "one artificial leg, made in New York, one in Boston, and one in Springfield, Mass., by *Palmer & Co.,*" inasmuch as Palmer & Co. never made a leg for him, Lipps, at either of those places.

We hope Dr. Bly will correct the statement made in the certificate by Mr. Lipps, and inserted in his Circular, as he cannot be willing to give currency to any statement so wholly incorrect.

Yours, PALMER & CO.

---

☞ All Physicians who desire to become subscribers to this Journal. both in this City and elsewhere, will please signify such wish by sending us the Name, Town, County and State, written as plainly as possible, so as to avoid mistakes. Those who do not thus favor us with this distinct understanding will receive only an occasional number, signifying *our* impression that they would do well to become *regular subscribers.*

